# El Paso County Medical Society

**Published:** 1900-11

**Authors:** 


### El Paso County Medical Society.

El Paso, Texas, October 25, 1900.

*To the Medical Profession of Texas, New Mexico, Arizona and Mexico.*

On the 13th day of October, 1900, the undersigned committee was appointed by the El Paso County Medical Society to invite the members of the regular medical profession of Texas, New Mexico, Arizona and Mexico to meet in El Paso, Texas, on the 17th day of January, 1901, for the purpose of organizing a Tri-State or Territorial Medical Association. (The El Paso Mid-Winter Carnival will be held January 17, 18, 19, 1901.) The committee hopes to have a large and enthusiastic meeting, that it may prove both pleasant and profitable, and they will expect every member of the profession throughout this territory to lend his personal efforts toward making the meeting a grand success.

Volunteer papers are requested from all who are willing to contribute, and you are especially requested to send in your name and the title of your paper at the earliest possible date, that the program may be completed and sent out in due time.

Will you kindly advise the committee of your intentions?

Reduced rates on all railroads.

S. T. Turner, M. D.,
W. N. Vilas, M. D.,
F. W. Galagher, M. D.,
Committee.

